# Towards a simple mathematical theory of citation distributions

**DOI:** 10.1186/s40064-015-1467-8

**Published:** 2015-11-05

**Authors:** Yurij L. Katchanov

**Affiliations:** National Research University Higher School of Economics (HSE), 20 Myasnitskaya Ulitsa, 101000 Moscow, Russia

**Keywords:** Bibliometrics, Citation distributions, Power law distribution, Wakeby distribution, 91D30, 91D99

## Abstract

The paper is written with the assumption that the purpose of a mathematical theory of citation is to explain bibliometric regularities at the level of mathematical formalism. A mathematical formalism is proposed for the appearance of power law distributions in social citation systems. The principal contributions of this paper are an axiomatic characterization of citation distributions in terms of the Ekeland variational principle and a mathematical exploration of the power law nature of citation distributions. Apart from its inherent value in providing a better understanding of the mathematical underpinnings of bibliometric models, such an approach can be used to derive a citation distribution from first principles.

## Background

Scholars have been investigating their own citation practice for too long. Bibliometrics already forced considerable changes in citation practice Michels and Schmoch (2014). Because the overwhelming majority of bibliometric studies focus on the citation statistics of scientific papers (see, e.g., Adler et al. 2009; Albarrán and Ruiz-Castillo 2011; De Battisti and Salini 2013; Nicolaisen 2007; Yang and Han 2015), special attention is devoted to citation distributions (see, inter alia, Radicchi and Castellano 2012; Ruiz-Castillo 2012; Sangwal 2014; Thelwall and Wilson 2014; Vieira and Gomes 2010). However, the fundamentals of the citation distribution (or CD for convenience) are far from being well established and the universal law of CD is still unknown (we do not go into details, and refer the reader to Bornmann and Daniel 2009; Eom and Fortunato 2011; Peterson et al. 2010; Radicchi et al. 2008; Ruiz-Castillo 2013; Waltman et al. 2012). Furthermore, existing bibliometric models of CDs place little or no emphasis on characteristics of the mathematical formalism itself (cf. Egghe 1998; Simkin and Roychowdhury 2007; Zhang 2013).

A mathematical theory of the CDs does not considers social citation system in its actuality. (We prefer to abbreviate social citation system to SCS; for the definition of SCS the reader is referred to, e.g., Fujigaki 1998; Rodríguez-Ruiz 2009; Rousseau and Ye 2012.) This task is completely left to scientometricists. The mathematical theory of CDs is used to investigate a mathematical substitute instead of a real process. For this mathematical substitute, the term mathematical structure has been introduced.

The objective of scientometrics is to bridge a gap between our insights of science and our knowledge of science Mingers and Leydesdorff (2015). A mathematical theory of citation can appear as an attempt to understand the structures that constitute the bases of scientometric models. To “understand” here means to bring a bibliometric structure into congruence with a mathematical structure. The purpose of a mathematical theory is fulfilled if it provides a structure of thought objects that allows us to relate bibliometric data sets and interpret the state of affairs in science by making mathematical deductions. A scientometric model attempts to create a heuristic explanation of an empirical data set. In contrast, a mathematical theory of citation is not concerned with bibliometric data per se and strives to construct a clear and coherent framework that accurately expresses some scientometric propositions in mathematical language. In this way, opportunities emerge for applying sophisticated mathematical concepts to bibliometric phenomena. The difference between a bibliometric model and a mathematical theory of citation is more apparent than real because, although the concepts of bibliometrics can be analyzed in terms of mathematics, they cannot be eliminated in favor of the latter without losing the understanding gained by bibliometrics. In particular, a firm foundation for a mathematical theory of citation can be obtained only phenomenologically by comparing the consequences of basic mathematical statements to bibliometric data.

### Motivation

We will study the axioms on which a mathematical description of SCS can be based. The author risks asserting that a mathematical theory appears to be a systematic reformulation of the problem of cumulative CDs on a purely mathematical basis. That is the main intent of this paper. Before we proceed with the analysis, we remark that there are no strong arguments leading from the bibliometric facts to the axioms. However, as we hope to show below, one can obtain additional conceptual information (relating to SCS) that is not readily available from a conventional bibliometric model by means of the axioms.

### Purpose

The purpose of the research reported in this article is to provide a simple and coherent presentation of CDs based on the Ekeland variational principle. We stress the elementary variational principle governing the state of SCS and have also attempted to provide enough technical detail to create a basis for potential future studies.

### Methodology

The paper addresses the construction of structural hypotheses for “how SCS works” rather than statistical inferences from bibliometric data. We accept that the continuous reproduction of a scientific inequality is a conceptual basis for almost all SCSs (cf. Bourdieu 2004). An emphasis is placed on the role of the variational principle as a valid approach for describing the local behavior of an continuous SCS. We consider an SCS to obey the following scheme. Suppose an SCS is a sufficiently smooth “motion” to ensure the consistency and the integrity of citations. In phase space, this condition is equivalent to a variational principle that produces the Euler equation for the weak form of a CD. This variational principle asserts that, for an appropriate functional, one can add a small perturbation to make it attain a minimum.

## Preliminaries

A mathematical theory of CDs cannot make sense of bibliometric models of CDs. However, this theory can make sense of mathematical models; therefore, it stipulate that bibliometrics must be presented in mathematical terms. We will call a function $z \mapsto N(z)$ giving the number *n* of scientific papers which have been cited a total of *z* times a *citation distribution* (CD). By construction, we define the event $\omega$ as the value of *z*, $\omega := z$. Under mild assumptions, it can be assumed (somewhat non-rigorously) that, with respect to the Lebesgue measure, for any Borel set *B*,

$$\begin{aligned} \bigl (\zeta (\omega )&=\omega \bigr )\bigl (\varOmega = [z_{\min }, z_{\max }]\bigr ): \mathsf {P}(\zeta \in B)&= \int _{B}f(z) dz \varpropto \int _{B}N(z) dz, \end{aligned}$$

$\zeta$ being a corresponding random variable (RV for short), defined on a certain probability space $(\varOmega , \mathcal {B}, \mathsf {P})$. Because of this result, without any great error one can also view the quantity *N*(*z*) as the probability density function (or, in abbreviated form, PDF) *f*(*z*) for finding the citation process at the point *z* in phase space (see also Redner 1998; Gupta et al. 2005), ignoring, for now, the objection that *z* and *n* assume only non-negative integer values. We do not consider the case where $\mathrm {supp} \,\, f(\cdot )$ is discrete separately. Although this is not mathematically rigorous, it is often useful to identify the CD $N(\cdot )$ with the PDF $f(\cdot )$ because a discrete SCS might be too complex to allow analytical results to be obtained.

All empirical CDs are different, but many have some statistical properties in common. In broad terms, power distributions of the form

1 $$\begin{aligned} (z \gg z_{\min }):f(z) \propto l(z) z^{- a} \end{aligned}$$

are frequently accepted without question. In the expression (1), $z_{\min }$ is a threshold value, and by *l*(*z*), we denote a slowly varying function (for the precise definition, see Borovkov 2013) such that for any fixed $k > 0$, the expression $\lim \frac{l(k z)}{l(z)}$, as $z\rightarrow \infty$, is equal to 1. More concretely, owing to complexities arising from the intricate citation dynamics of papers, the age distribution of references, the role of scientific journals, etc., the CDs are quite complicated in detail. However, to a reasonable approximation, a CD can be represented (in the long-time limit of the observation period) by the relation (1) (among an abundant literature, we refer to Albarrán et al. 2011; Brzezinski 2015; Egghe 2005; Radicchi and Castellano 2015; Redner 2005; Wallace et al. 2009). The systematic study of CDs’ deviations from power laws is not the subject of this paper. However, the literature on this topic is currently growing; the reader can see, e.g., Golosovsky and Solomon (2012), Golosovsky and Solomon (2014); Radicchi et al. 2012), Thelwall and Wilson (2014), Wang et al. (2013) and Yao et al. (2014).

Through the paper, we adopt the following notation: *V* is a real separable reflexive Banach space equipped with a norm $\Vert \cdot \Vert$, and $V^{\star }$ is its topological dual endowed with the natural norm $\Vert \cdot \Vert _{\star }$. The duality mapping between *V* and $V^{\star }$ is denoted by $\langle \cdot , \cdot \rangle$. In addition, recall that for a nontrivial function $\phi (\cdot ):V\rightarrow \mathbb {R}\cup \lbrace +\infty \rbrace$ with the effective domain

$$\begin{aligned} \mathrm {dom}\,\phi := \bigl \lbrace v \in V :\phi (v) < +\infty \bigr \rbrace , \end{aligned}$$

the subset of $V^{\star }$

$$\begin{aligned}(\forall v\in V):\partial \phi (v_{0}) = \left\{ v^{\star }\in V^{\star }:\phi (v) - \phi (v_{0})\ge \bigl \langle v^{\star }, v - v_{0}\bigr \rangle \right\} \end{aligned}$$

is said to be the subdifferential of $\phi (\cdot )$ at $v_{0}$.

In the language of $\mathsf {P}(Z \in B)$, the PDF $f(\cdot )$ is (almost everywhere) given by formal differentiating; as a result of this, a rather simple interpretation of $f(\cdot )$ can be given in the framework of Sobolev spaces $H^{k}(\mathbb {R})$. (For the definitions and properties of Sobolev spaces, see Maz’ya 2011.)

Unless specified, in the following, *I* is an open interval in $\mathbb {R}$. For technical reasons, $H^{k}(I)$ is a good example of the space of RVs $\zeta$ such that

$$\begin{aligned} \left( \forall \zeta \in H^{k}(I)\right) :\int _{I}|\zeta |^{k} \mathsf {P}(dz) < \infty . \end{aligned}$$

In addition to the probabilistic treatment, one can say that an SCS acting on some function $\varphi (\cdot )$ yields an RV $\zeta$. In other words, we can also state that an SCS allow us to bring one and only one well-defined RV $\zeta \in V$ into correspondence with each function $\varphi (\cdot )\in V^{\star }$.

## Results

Because $\varphi (\cdot )$ and $\zeta$ are so fundamental in this paper, it may seem strange that we have not explicitly defined them in formal mathematical terms. As with other primitive objects of the mathematical theory, the most one can do is to give the implicit definitions by postulating the properties that hold for $\varphi (\cdot )$ and $\zeta$.

We shall attempt now to shed some light upon the relation between the function $\varphi (\cdot )$ and the quantity $\zeta$. Pick any $\varepsilon > 0$; then, the partial order of Bishop – Phelps on $V\times \mathbb {R}$ can be defined as follows (cf. Johnson and Lindenstrauss 2001):

2 $$\begin{aligned} \left[ v_{1},v_{1}^{\star }\right] \preceq \left[ v_{2},v_{2}^{\star }\right] \Longleftrightarrow \left\langle v_{1}^{\star }, v_{1}\right\rangle + \varepsilon \Vert v_{1} - v_{2}\Vert \le \left\langle v_{2}^{\star }, v_{2}\right\rangle . \end{aligned}$$

For a nonempty closed convex subset $M\subset V\times \mathbb {R}$ which is bounded below, in sense that,

$$\begin{aligned}&\left( v^{\star }\in V^{\star }\right) : \inf _{V} \Bigl \lbrace \left\langle v^{*},v\right\rangle \in \mathbb {R}:\exists v\in V, \ \left[ v, v^{\star }\right] \in M\Bigr \rbrace > - \infty , \end{aligned}$$

there is a minimal element $[v_{*}, v_{*}^{\star }]$ in the partial order (2), according to the classical theorem of Bishop – Phelps (see, e.g., Deville and Ghoussoub 2001). In this connection, it is evident that the map $\varphi \mapsto \zeta$ is nothing but the Riesz – Fréchet isomorphism from $V^{\star }$ onto *V*. This means that we have characterization via

$$\begin{aligned} \bigl (\zeta , v\bigr )_{V} = \bigl \langle \varphi , v \bigr \rangle _{V^{\star },\, V} , \end{aligned}$$

where $(\cdot ,\cdot )_{V}$ indicates the scalar product in *V*. Based on this assertion, we will collect the basic properties of $\varphi (\cdot )$ and $\zeta$ in the following axioms:

$$\mathbf {A_{1}}$$
A function $\varphi (\cdot )$ for $\forall v \in \bigl ( V, \Vert \cdot \Vert \bigr )$ is a proper ($\mathrm {dom}\,\varphi \not = \emptyset$), lower semicontinuous ($\varphi (v)\le \liminf _{n\rightarrow \infty }\varphi (v_{n})$ if $v_{n}\rightarrow v$), convex, and bounded below ($\inf _{V}\varphi > -\infty$) function from $\bigl (V, \Vert \cdot \Vert \bigr )$ to $\mathbb {R}_{+}$, satisfying the following condition:
  $$\begin{aligned}(\forall v\in V)(\forall c\in \mathbb {R}):\varphi (u+v)=\varphi (u)+\varphi (v)\Leftrightarrow v= |c|u. \end{aligned}$$
$$\mathbf {A_{2}}$$
Among all admissible $\zeta$, the quantity $\zeta _{*}$ which actually describes a given CD, is assigned in such a way that the function $\varphi (\cdot )$ reaches its minimum.

The axiom $\mathbf {A_{1}}$ hinges on a corollary Aubin and Ekeland (2006), p. 262 of the Ekeland variational principle Ekeland (1974). The term “Ekeland variational principles” refers here essentially to a result stating that the function $\varphi (v)$ possesses arbitrarily small perturbations

$$\begin{aligned} (v \in \mathrm {dom}\,\varphi )(\varepsilon > 0):\varphi (v)\le \inf _{V}\varphi + \varepsilon , \end{aligned}$$

such that the perturbed function

$$\begin{aligned} \bigl (\forall v \in V \backslash \lbrace v_{\varepsilon }\rbrace \bigr ):v \mapsto \varphi (v) + \varepsilon \Vert v - v_{\varepsilon }\Vert , \end{aligned}$$

will have an absolute (and even strict) minimum Ioffe and Tikhomirov (1997). More precisely, there exists $v_{\varepsilon }\in \mathrm {dom}\,\varphi$ and $v^{\star }_{\varepsilon }\in \partial \varphi (v_{\varepsilon })$ such that

$$\begin{aligned}&\bigl (\forall v \in V \backslash \lbrace v_{\varepsilon }\rbrace \bigr ):\left\| v - v_{\varepsilon }\right\| \le \varphi (v) - \varphi (v_{\varepsilon }), \\&\bigl (\partial \varphi (v_{\varepsilon }) \not = \emptyset \bigr ):\left\| v^{\star }_{\varepsilon } \right\| _{\star } \le \varepsilon . \end{aligned}$$

Loosely speaking, if $v_{\varepsilon }$ is at least as good as *v*, then $v_{\varepsilon }$ is almost the same function for which the minimum of $\varphi (\cdot )$ is almost achieved. At the same time, it is important to stress that the Ekeland variational principle does not guarantee that $\varphi (\cdot )$ attains its minimum.

The essential features of our approach include the use of Sobolev spaces. Consider now the Gelfand triple

$$\begin{aligned} V = \widetilde{H}(I) \hookrightarrow L^{2}(I) \hookrightarrow \widetilde{H}^{-1}(I) = V^{\star }, \end{aligned}$$

where

$$\begin{aligned} \widetilde{H}(I) := \left\{ v, v_{\varepsilon } \in H^{1}(I):v - v_{\varepsilon } \in H^{1}_{0}(I)\right\} \end{aligned}$$

is a closed affine subspace of the second-order Sobolev space $H^{1}(I)$ (which is also a separable Hilbert space) and the embeddings are dense, continuous, and compact. Given a function $v_{\varepsilon }$, we set

$$\begin{aligned} \left( \forall v, v_{\varepsilon } \in H^{1}(I)\right) :\tilde{v} := v - v_{\varepsilon }. \end{aligned}$$

From now on, we will use the transformed functions $\tilde{v}$, but for convenience drop the tilde.

The space $V:= \widetilde{H}(I)$ is endowed with the usual scalar product

$$\begin{aligned} \left( \forall u, v \in V\right) :(u, v)_{V} = \int _{I}\left( uv + u^{\prime }v^{\prime }\right) . \end{aligned}$$

We see that the associated norm

$$\begin{aligned} \Vert v\Vert ^{2}_{V} = \int _{I}\left( v^{\prime 2} + v^{2}\right) \end{aligned}$$

satisfies the axiom $\mathbf {A_{1}}$. Before moving on to consider $\zeta$, however, it is tempting to slightly generalize the definition of *V*. Modelization of an complex real SCS requires us to introduce some additional constructions. The differential operator *K*,

$$\begin{aligned}&\left( 0<\alpha \le p(\cdot )\in C^{1}\bigl ( \overline{I} \bigr )\right) \left( 0\le q(\cdot )\in C\bigl ( \overline{I} \bigr )\right) \\&\left( \forall v \in \widetilde{H}(I)\right) : v:= -\bigl ( p(\cdot ) v^{\prime } \bigr )^{\prime } + q(\cdot ) v, \end{aligned}$$

is an isomorphic map $K:V \leftrightarrows V^{\star }$. We will work on the so-called energetic space $H_{E}(I)$ such that

$$\begin{aligned}&V_{E} = {H_{E}(I)}\hookrightarrow {\widetilde{H}(I)} \hookrightarrow {L^{2}(I)} \hookrightarrow {\widetilde{H}^{-1}(I)} \hookrightarrow {H_{E}^{-1}(I)} = V_{E}^{\star }. \end{aligned}$$

Hereafter we use the notation ${E}:= V_{E} = H_{E}(I)$. To avoid overloading our presentation, we refer the reader to Zeidler (1999) for details, proofs and explanations; the interested reader can compare this approach to the one described in Kristály et al. (2010).

Thanks to the notation introduced above, the energetic space *E* is equipped with the scalar product given by

3 $$\begin{aligned}\left( \forall \tilde{u}, v \in E\right) :(u, v)_{E} = \int _{I} \left( p u^{\prime } v^{\prime } + q u v\right) . \end{aligned}$$

The induced energetic norm can be written as

$$\begin{aligned} \bigl (\forall v \in E\bigr ):\Vert v \Vert _{E}^{2} = \int _{I}\left( p v^{ \prime 2} + q v^{2}\right) . \end{aligned}$$

By definition, put

4 $$\begin{aligned} \bigl (\forall v \in E^{}\bigr ):\varphi (v) = \Vert v \Vert _{E}^{2}. \end{aligned}$$

That is, $\varphi$ may be interpreted as measuring the average value of the weak derivatives. Recall from Attouch et al. (2014) that the statements listed below are equivalent.

$$S_{1}$$
 There exists a unique $\zeta \in E$ such that
  5 $$\begin{aligned} \bigl (\forall v\in E \bigr ):\bigl ( \zeta , v \bigr )_{E} = 0. \end{aligned}$$
  The formula (5) reads as the “weak” Euler equation in the current setting.
$$S_{2}$$
 $\zeta$ is obtained by
  6 $$\begin{aligned} \min _{v\in {E}}\left\{ \frac{1}{2}\,\varphi (v) \right\} . \end{aligned}$$

It is well known from the theory for variational problems in Sobolev spaces that any local minimizer of $\varphi (v)$ in the *E* topology is also a local minimizer of $\varphi (v)$ in the $C^{1}$ topology, and it follows in the standard way that for the quantity $\zeta$, we have

7 $$\begin{aligned} \zeta = \lambda \left( c_{1}\cosh \eta + c_{2}\sinh \eta \right) , \end{aligned}$$

where

8 $$\begin{aligned} \eta&= \int \left( \frac{q(z)}{p(z)}\right) ^{\frac{1}{2}} dz ,\end{aligned}$$

9 $$\begin{aligned} \lambda&= \bigl (p(z) q(z)\bigr )^{-\frac{1}{4}} , \end{aligned}$$

and $c_{1}$, $c_{2}$ are constants.

To arrive at specific, relevant RV $\zeta$ one has to make an assumption in addition to the axioms $\mathbf {A_{1}}$ and $\mathbf {A_{2}}$. Now let us choose the function $z\mapsto \eta$ defined in Eq. (4) according to the formula (1), i.e. in the form of a slowly varying function (see Borovkov 2013). Therefore, we have

$$\begin{aligned} \lim _{z\rightarrow \infty } \eta (z) = o(\ln z). \end{aligned}$$

We then introduce the function $\eta (z)$ given by

10 $$\begin{aligned} \eta (z) \propto \ln z . \end{aligned}$$

To set up the problem, we eliminate the factor $\lambda$ from Eq. (7) by rescaling the quantity $\zeta$

11 $$\begin{aligned} (\lambda )^{-1}\zeta \rightarrow \zeta . \end{aligned}$$

In an appropriate normalization, instead of the original RV $\zeta$ occurring in Eq. (7), we now have to deal with the renormalized RV $\zeta$. Substituting expressions (11) and (12) into Eq. (8), we obtain

12 $$\begin{aligned} \zeta \propto \kappa _{1} z^{-\delta } + \ \kappa _{2} z^{\beta }. \end{aligned}$$

At least for all practical purposes, it is possible to represent the relation (12) by means of a standard uniform RV *U*

13 $$\begin{aligned} \zeta \propto \kappa _{1} U^{-\delta } + \ \kappa _{2} U^{\beta }. \end{aligned}$$

We will treat Eq. (13) in a broader sense — as the Wakeby distribution (WD) (for more details, see Katchanov and Markova 2015; the WD features may be found in Hosking and Wallis 2005). Introducing the continuous parameters $\beta$, $\gamma$, $\delta$, which are called shape parameters in statistics, the continuous location parameter $\xi$, and the continuous scale parameter $\alpha$, we may cast Eq. (13) into the following general WD form Johnson et al. (2010), p. 44–46

14 $$\begin{aligned} \zeta = \xi +\frac{\alpha }{\beta }\Bigl (1 - (1 - U)^{\beta }\Bigr ) - \frac{\gamma }{\delta }\Bigl (1 - (1 - U)^{-\delta }\Bigr ). \end{aligned}$$

In a special, but very important case, when $\alpha = 0$ or $\gamma = 0$, the WD in Eq. (14) reduces to the generalized Pareto distribution (GPD)

15 $$\begin{aligned} \zeta = \xi - \frac{\gamma }{\delta }\Bigl (1 - (1 - U)^{-\delta }\Bigr ). \end{aligned}$$

**Fig. 1:**
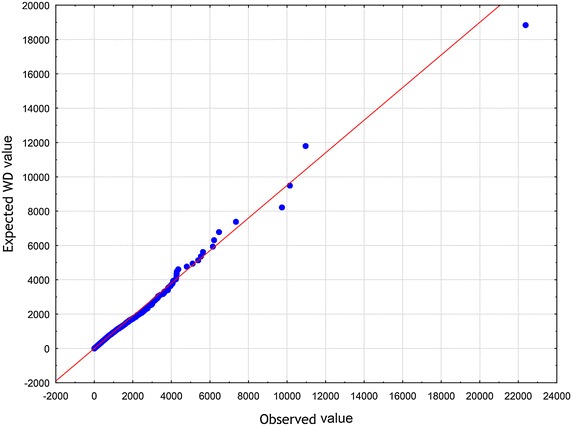
WD Q–Q plot of *z*

**Fig. 2:**
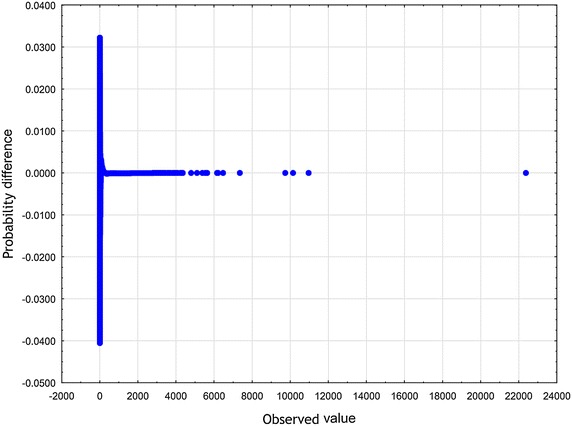
WD probability difference plot of *z*

**Fig. 3:**
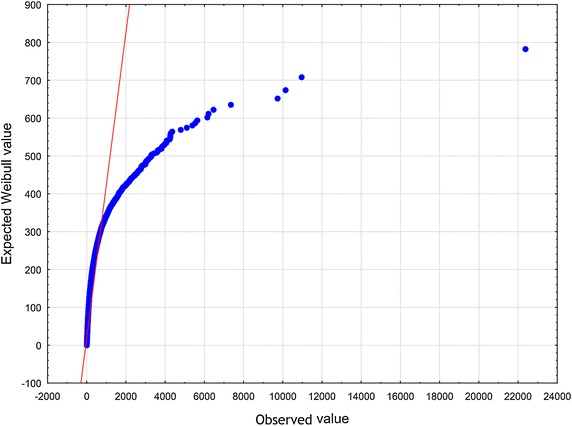
Weibull distribution Q–Q plot of *z*

**Fig. 4:**
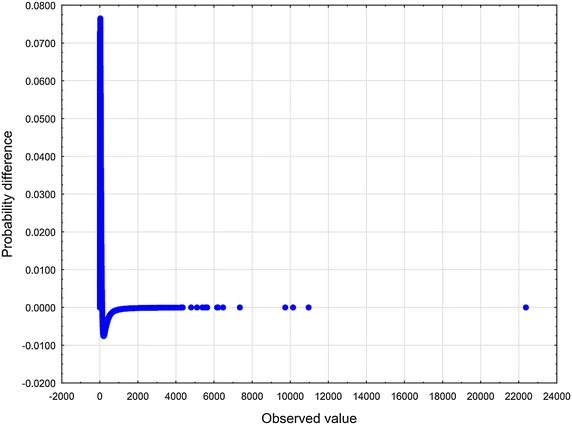
Weibull distribution probability difference plot of *z*

**Fig. 5:**
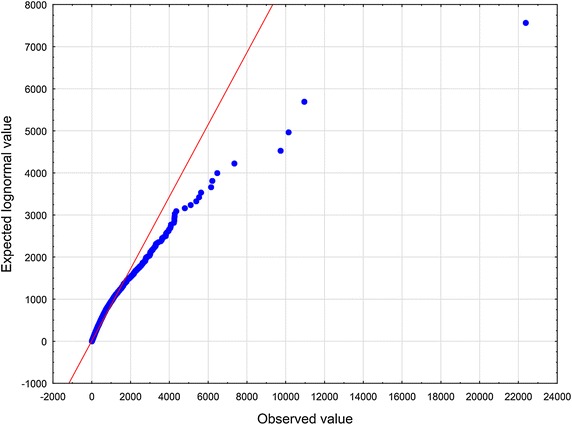
Lognormal distribution Q–Q plot of *z*

**Fig. 6:**
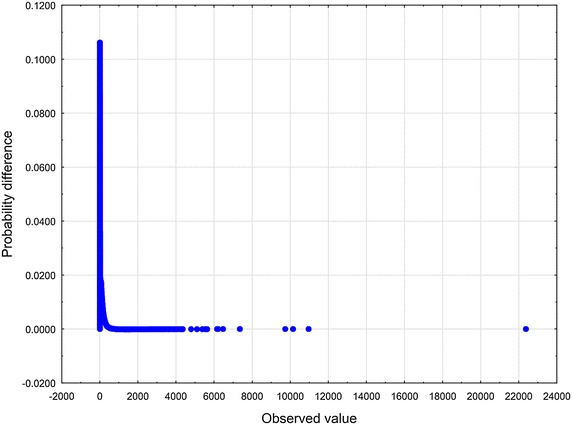
Lognormal distribution probability difference plot of *z*

To furnish a concrete illustration, we collected the sample of articles and reviews published in journals that put in print more than 100 documents per year and were indexed in Journal Citation Reports 2003 Science edition (Thomson Reuters). All data were downloaded from Web of Science (WoS, updated on August 8, 2013), with a 10-year time window. The number of citations *z* was counted as the total number of times a paper appears as a reference of a more recently published paper indexed in the Web of Science Core Collection. There are 31, 097, 160 citations among 1, 062, 961 papers. The WD, the Weibull, and the lognormal distribution were fitted these bibliometric data using the ‘lmomco’ R package. Goodness-of-fit was done based on Kolmogorov – Smirnov’s statistic *D* and Anderson – Darling’s statistic *A*. The values of the test statistics are reported in Table 1 (see also Figs. 1, 2, 3, 4, 5 and 6). Comparing the obtained values and goodness-of-fit statistics given in the Table 1, it will be seen that the WD offers a higher level of accuracy than the other probability distributions considered. We conclude that the CD obtained turns out to be similar to the WD.

**Table 1:** Goodness of fit—summary

Distribution	Kolmogorov–Smirnov ${\alpha = 0.1} ,$ Crit. val. 0.0013 Statistic *D*	Anderson–Darling ${\alpha = 0.1} ,$ Crit. val. 1.929 Statistic *A*
WD	0.0406	1449.0
Lognormal	0.1062	$1{.}258\mathrm {E}+5$
Weibull	0.1262	$1{.}319\mathrm {E}+5$

## Discussion

One of the most exciting and fruitful applications of mathematical methods in the natural sciences is the variational principle. The substantive aim of the present paper is the derivation of a variational principle, which makes it possible to interpret the empirical regularities of the CDs as a logical necessity. Starting from the famous Ekeland variational principle, we show that the derivation of the CDs given in this paper might be considered a step in indicated above direction. Using the variational principle (6) in the energetic space *E* together with empirical evidences about the existence of the slowly varying functions representing the right tail of the CDs allows us to introduce the WD (and the GPD) naturally.

Let us stress that modest mathematical means concerning some simple facts of functional analysis yield a simple mathematical theory of CDs from which, as its consequence, concrete CDs are immediate derived. It is remarkable that a first-principles derivation of the CDs (e.g., GPD) in a bibliometric model is possible at the price of uncontrollable assumptions, which are justified a posteriori. On the contrary, in our derivation it is only assumed that Eq. (8) is relevant. This is, of course, more satisfactory. However, note that there are no proper bibliometric reasons for which the Sobolev spaces are preferred over any other, and, therefore there are also no reasons to give the vague bibliometric meaning of the consistency and the integrity of citations the mathematical form of Ekeland’s variational principle.

One must bear in mind that our result refers to properties of some “pure mathematical structure”. Like any mathematical result, the Eq. (13) cannot give a completely accurate description of a empirical CD. Moreover, in the mathematical theory of CDs, “by construction”, we have no direct knowledge of the statistical parameters. Thus, we can only measure the parameters that index the CDs, not compute them from the axioms.

## Conclusions

In summary, the approach suggested here allows an interpretation of the Ekeland variational principle in terms of the standard uniform RV, which may have some interest. It is shown that in a sufficiently “smooth” SCS a power-law tail of the static CD can appear. However, there are no grounds to consider this a mathematical model underlying bibliometric theory. At the same time, the present study may be instructive beyond the specific research site and can contribute to a mathematical theory of CDs building.
